# End‐user preference for and choice of four vaginally delivered HIV prevention methods among young women in South Africa and Zimbabwe: the Quatro Clinical Crossover Study

**DOI:** 10.1002/jia2.25283

**Published:** 2019-05-09

**Authors:** Elizabeth T Montgomery, Mags Beksinska, Nyaradzo Mgodi, Jill Schwartz, Rachel Weinrib, Erica N Browne, Nonhlanhla Mphili, Petina Musara, Manjeetha Jaggernath, Susan Ju, Jenni Smit, Z Mike Chirenje, Gustavo F Doncel, Ariane van der Straten

**Affiliations:** ^1^ Women's Global Health Imperative RTI International San Francisco CA USA; ^2^ MRU (MatCH Research Unit) Faculty of Health Sciences University of the Witwatersrand Durban South Africa; ^3^ University of Zimbabwe College of Health Sciences Clinical Trials Research Centre Harare Zimbabwe; ^4^ CONRAD Eastern Virginia Medical School Arlington VA USA; ^5^ Center for AIDS Prevention Studies Department of Medicine University of California San Francisco CA USA

**Keywords:** microbicides, PrEP, adolescents, women, South Africa, Zimbabwe

## Abstract

**Introduction:**

Adherence to HIV prevention methods is a challenge, particularly for young women in Sub‐Saharan Africa. End‐user research during product development can inform modifiable factors to increase future uptake and adherence.

**Methods:**

Preferences for four vaginally inserted placebo HIV prevention methods were assessed among Zimbabwean and South African young women using a crossover clinical design. For each of months 1 to 4, participants were asked to use a pre‐coitally inserted film, insert (vaginal tablet) and gel once/week for a month, and a monthly ring in a randomly assigned sequence. Participants subsequently chose one preferred product to use as directed for the final study month. Women ranked the four products from most preferred to least preferred at enrolment and after trying all products.

**Results:**

A total of 200 women aged 18 to 30 (mean 23) were enrolled; 178 (89%) completed follow‐up. At baseline, 41% of participants selected the gel as their most preferred product and 61% selected the ring as least preferred. During the crossover period, most (82% to 85%) self‐reported using each product at least once a week, although only half the time with sex. Objective biomarker data confirmed adequate use of all products. After trying each product, rankings changed with the film, ring, insert and gel being selected by 29%, 28%, 26% and 16% respectively. Choice varied significantly by country (*p *<* *0.001): More Zimbabweans chose the film (45%), and more South Africans chose the insert (34%). Among women choosing the ring, 88% reported using it every time with sex. By contrast, self‐reported adherence was lower for “on‐demand” (coitally associated) products, with 40% to 55% using them every time during sex (*p *<* *0.001).

**Conclusions:**

Preferences for these four dosage forms varied before and after use, and both within and across countries – there was no clear favourite – indicating the need for a range of options for end‐users The ring's popularity increased the most with use, was the second most preferred delivery system, and per self‐report, provided more coverage during sex. These end‐user perspectives provide important information to product developers and funding agencies.

## 1 Introduction

Adolescents and young women in Africa are at disproportionately high risk for HIV acquisition and the identification and delivery of safe, effective female‐initiated prevention interventions is a public health priority 1. Female condoms and oral pre‐exposure prophylaxis (PrEP) are currently the only licensed and available biomedical interventions that young women could use autonomously to prevent HIV. Female condoms have the advantage of also preventing pregnancy and other STIs, but have had mixed acceptability, are not discrete and are not widely available 2, 3. Oral PrEP is currently approved in a handful of Sub‐Saharan African countries, but only available in limited delivery settings 4. Furthermore, it must be prescribed and taken daily and requires regular clinic safety monitoring, which is a challenge for, and undesired by, many users 5. More recently, the monthly dapivirine ring was proven safe and partially effective at preventing HIV among African women in two large trials 6, 7, and open‐label extension studies and an application for licensure are currently underway 8. Nevertheless, biomarkers of adherence and qualitative data indicate that some women do not consistently use the ring, in particular young women. Consistent with previous trials of candidate microbicide gels and cervical barriers, ring nonadherence may have been caused by various contextual reasons such as perceived interference with sex, menses, short‐term periods of abstinence and nondisclosure to male partners 9, 10, 11, 12, 13, 14. As with oral PrEP, and other antiretroviral‐based approaches in development, use of a licensed dapivirine ring would require regular clinic visits and monitoring.

In response to challenges with suboptimal adherence in HIV prevention trials, research with future end‐users during product development is recognized as a priority so as to identify preferences that might ultimately improve future uptake and adherence. The overall goal of the “Quatro” Study, a name alluding to “four” in romance languages, was to assess young African women's use experiences and preferences in regard to four vaginally inserted placebo dosage forms that are in development as drug delivery mechanisms for HIV and multipurpose indications: a ring, film, vaginal tablet and gel. While both the ring and the gel have been tested in later stage human trials 6, 7, 15, the vaginal film and tablet are in preclinical or phase 1 testing 16. This study elicits comparative feedback from young African women – a critical end‐user population – of multiple dosage forms that are at varying stages of product development, and provides important information about what prevention methods women would choose to use.

## 2 Methods

A randomized crossover design assessed relative preferences of four vaginal placebo dosage forms. Participants were asked to use pre‐coitally inserted film, insert (vaginal tablet) and gel once per week for a month, and a monthly ring (for the entire month) during a four‐month crossover period. Participants subsequently chose one preferred product to use as directed for the fifth and final study month. Participants were enrolled and followed between June 2016 and June 2017 at two clinical sites in Chitungwiza, Zimbabwe, and Durban, South Africa. All procedures were reviewed and approved by ethics and regulatory bodies in the US (Chesapeake IRB: 00150063), South Africa (Human Research Ethics Committee of the University of the Witwatersrand (151106); Medicines Control Council (DB: N2/19/8/2)) and Zimbabwe (Medical Research Council of Zimbabwe (MRCZ/A/1988), Medicines Control Authority of Zimbabwe (B/279/5/07/2016)) before implementation.

### 2.1 Study setting and population

In Chitungwiza, Zimbabwe's second most populous city 20 km from the capital of Harare, women were recruited from municipal clinics, HIV testing centres, shopping centres and markets, surrounding farming communities and informal settlements and through health worker and participant referrals. In Durban, women were recruited from a reproductive health clinic attached to the research site in a centrally located urban city centre and from the local community via the research site outreach team. HIV‐negative, nonpregnant, sexually active women (defined by heterosexual vaginal intercourse at least four times per month in the past three months) ages 18 to 30 were eligible to enrol. Women who had participated in prior HIV prevention product trials or demonstration studies were excluded, and those with symptomatic STIs, pelvic exam findings or other genitourinary complaints at screening were referred for treatment and were allowed to enrol after symptoms cleared. The study adopted a novel co‐designer approach that engaged women to consider their opinions as critical input regarding product attributes throughout participation, and emphasized the need for candid and honest feedback about likes and dislikes of products, use/nonuse of products and suggestions for design modifications. In addition to training staff to employ this approach towards participants, the role of co‐designer was instilled through recruitment messages and fliers that used the “co‐designer” terminology; ongoing engagement throughout the study including bimonthly participant meetings and events, and presentation of co‐designer certificates at the end of study participation.

### 2.2 Study products

**Figure 1:**
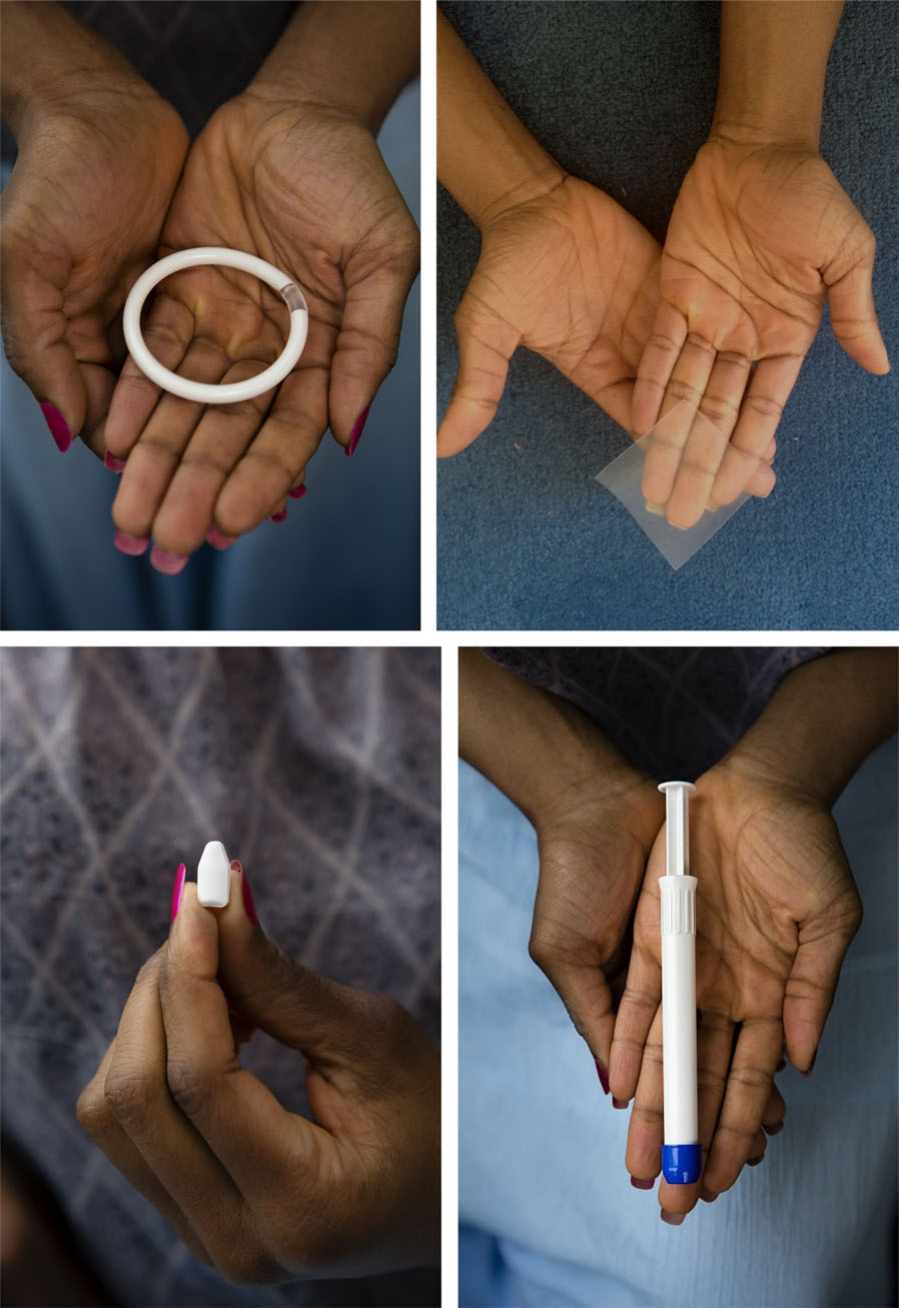
Quatro study products, all placebo. Clockwise from top left: Polyurethane vaginal ring, vaginal film, vaginal gel (inside applicator), vaginal insert.

Four vaginal study products developed by CONRAD, Eastern Virginia Medical School (Arlington, VA) were investigated: gel (hydroxyethyl cellulose (HEC), manufacturer: DPT Laboratories, San Antonio, TX), a polyurethane ring (manufacturer: Particle Sciences, Inc., Bethlehem, PA), a film (provided by Magee‐Womens Research Institute and Foundation, manufacturer: Par Pharmaceutical, Woodcliff Lake, NJ) and a tablet‐like vaginal insert (manufacturer: CoreRx) (See Figure 1). Placebo products were used because the study objective was to explore and compare acceptability of the delivery forms in early stages of development, irrespective of the active ingredients.

### 2.3 Procedures

Following an informed consent process, participants were screened for inclusion using a brief clinical questionnaire to assess general health, a pelvic exam to rule out genitourinary issues that could interfere with or cause discomfort during product use, and laboratory testing for HIV infection and pregnancy. At baseline, enrolled participants answered a detailed questionnaire that included questions about sexual behaviour, HIV risk perception, prior knowledge and perceptions of HIV prevention products and their opinions of the four study products before and after watching a brief animated video that introduced the products in a standardized way. To minimize bias from an order effect, participants were randomized to receive the four products in one of the 24 possible sequences, and dispensed the first of four assigned products and its corresponding usage instructions. During the four‐month cross‐over period, participants first inserted their product in the clinic as practice under direct observation. For each of the three on‐demand products (insert, film and gel), women were dispensed five doses to use at home and were instructed to insert them once per week within two hours before having sex, or, if use during sex was not an option, to use the product anyway without sex. Women were instructed to use the ring for the entire month. The objective of the cross‐over period was to provide sufficient exposure to products to adequately inform opinions and preferences.

In addition, four vaginal swabs were dispensed at enrolment and each follow‐up visit to collect biomarker data for post‐trial adherence assessment (data to be published elsewhere). Participants practiced self‐swabbing in the clinic and were instructed to self‐swab at home either immediately after having sex with the product, or, if the product was used without sex, approximately two hours after inserting the product or once a week if using the ring. Each month, women returned to the clinic and received risk‐reduction counselling, a clinical assessment and administration of interviewer‐administered acceptability questionnaires. Women returned unused on‐demand products, used rings and vaginal swabs; collected their next assigned product; and were reminded that the products were placebos and provided with condoms. Participants were provided with a phone number for the study cell phone and advised to call the study at any time to discuss product use, visit scheduling, or other issues; and to visit the clinic at any time if there were clinical concerns or products needed replenishment. At the study exit, HIV and pregnancy tests were repeated. For their time and travel, participants were reimbursed an amount commensurate with local IRB requirements at all study visits.

After trying all four products during the crossover period, participants chose a product to use for the final study month (Month 5). Participants who selected one of the three on‐demand products were instructed to use the product every time they had sex. If they selected the ring, they were instructed to leave it inserted for the entire month. The objective of the use period was to assess participants’ acceptability and use of their chosen product, when using it consistently and during sex.

### 2.4 Measures

Outcome measures included product ranking, future consideration, choice and use. For ranking, participants were asked to compare each of the four products to each other in a ranked order, from one to four. Rankings were measured at baseline before and after watching the educational video, after using all four products (Month 4), and at study exit. At Month 4, participants were asked about their future willingness to use each product. Interviewers asked *of the four products, are there any products you would not consider using in the future? You can select more than one*, followed by naming each of the four products. The participant could voluntarily indicate she would consider using all. “Choice” was defined as the product the participant chose to use during Month 5. Product use, with and without sex, was measured at every follow‐up visit using women's self‐report of behaviour over the past month. During the cross‐over period, product use was intended to contextualize women's opinions with their level of exposure to the products and their use during sex.

### 2.5 Statistical analysis

For our outcome of product choice, a sample of 200 was estimated to have adequate statistical power (≥0.80) to detect a difference in proportion of 0.20, with two‐sided alpha of 0.05; this was assuming the proportion choosing one of the products was between 0.30 and 0.40 and another between 0.10 and 0.20. A 20% difference in choice of a product was considered clinically meaningful. Chi‐square tests were used to compare differences in participants’ baseline characteristics by country. Mixed‐effect logistic regression models were used to estimate the proportion ranking each product first (#1) and last (#4) from a product‐naïve state at baseline, after receiving standardized information about the products through the brief educational video, and after having the opportunity to try the product for one month. The models controlled for country and included a random participant effect to account for the longitudinal structure of the data. For each product, we used a logistic regression model to explore associations between each baseline characteristic and choice of product at Month 5, controlling for country and crossover month of use. *p*‐values presented were not adjusted for multiple comparisons; a value of *p *<* *0.05 was considered significant. All analyses were conducted using Stata 15.0 (StataCorp LLC, College Station, Texas).

## 3 Results

### 3.1 Study sample

In total, 257 women were screened and 200 enrolled into the clinical study; 180 (90%) completed the crossover period and 176 (88%) completed the study. Women lost to follow‐up did not differ from those who completed the study on key demographic characteristics examined or randomization group (*p *≥* *0.05, data not shown).

Table 1 describes the characteristics of the study population. The median age was 24 years (interquartile range 21 to 26), and the majority (70%, n = 139) had completed secondary school. Nearly all (94%) Zimbabwean women were married or living with their partner, compared to 10% in South Africa. All women in Zimbabwe, and 72% in South Africa, were parous (ranging from 1 to 4). Women in Zimbabwe were more likely to earn an income (52% vs. 15%), and South African participants reported more food insecurity. Participants in South Africa were more likely than Zimbabweans to have ever used injectable contraceptives (71% vs. 33%), and this was the most commonly used contraceptive in South Africa. In Zimbabwe most participants (86%) had used pills, and almost half (49%) had used an implant. No one reported contraceptive ring use in these study settings.

**Table 1:** Characteristics of participants enrolled in the Quatro study, overall and by country

	Durban, South Africa (N = 100) %	Harare, Zimbabwe (N = 100) %	Total (N = 200) %	*p*‐value
Age, years				
Median (IQR)	23 (20 to 26)	24 (22 to 26)	24 (21 to 26)	0.06
18 to 24	67	54	61	
25 to 30	33	46	40	
Currently have a primary partner	94	100	97	0.01
Live with partner or married	10	94	52	<0.001
Currently have a casual sex partner	8	2	5	0.05
Ever exchanged sex	3	2	3	0.65
Parity > 0	72	100	86	<0.001
Completed secondary school	76	63	70	0.05
Earns an income	15	52	34	<0.001
Attend religious services at least once a week	69	100	85	<0.001
No food insecurity past 4 weeks	50	86	68	<0.001
Worried contract HIV in next 12 months				
Not at all/a little	51	46	49	0.48
Somewhat/very/extremely	49	54	52	
Place for privacy in home	91	96	94	0.15
Any intravaginal practices past 3 months (inserted something for menstrual control or to clean/tighten/dry vagina)	82	91	87	0.06
Contraceptive methods ever used				
Male condom	93	59	76	<0.001
Female condom	1	7	4	0.03
Pills	30	86	58	<0.001
IUD	3	2	3	0.65
Implants	15	49	32	<0.001
Injectable	71	33	52	<0.001

During the five‐month follow‐up period two incident HIV infections were identified among the South African participants, as well as five pregnancies: three in South Africa, two in Zimbabwe. No serious adverse events associated with study products were reported. There were 30 ring‐related, 3 insert‐related, 17 film‐related and 5 gel‐related adverse events (Table 2). There was one social harm reported; the participant said her partner was refusing to have sex with her because she was using the ring.

**Table 2:** Product‐related adverse events in the Quatro study (June 2016‐June 2017)

Adverse event	Ring		Insert		Film		Gel	
	N	Rate per person‐months	N	Rate per person‐months	N	Rate per person‐months	N	Rate per person‐months
Vaginal discharge	14	0.06	1	<0.01	4	0.02	2	0.01
Lower abdominal pain	6	0.03	0	0.00	3	0.01	1	<0.01
Vaginal rash	1	<0.01	1	<0.01	2	0.01	0	0.00
Dysuria	2	0.01	0	0.00	2	0.01	0	0.00
Intravaginal pain	3	0.01	0	0.00	0	0.00	0	0.00
Vaginal pruritus	1	<0.01	0	0.00	1	<0.01	1	<0.01
Nausea	1	<0.01	0	0.00	1	<0.01	0	0.00
Dyspareunia	2	0.01	0	0.00	0	0.00	0	0.00
Skin rash/urticaria	0	0.00	0	0.00	1	<0.01	1	<0.01
Vaginal paresthesia	0	0.00	1	<0.01	1	<0.01	0	0.00
Abnormal vaginal bleeding (spotting)	0	0.00	0	0.00	2	0.01	0	0.00
Total events	30	0.13	3	0.01	17	0.07	5	0.02
Total person‐months	236		231		239		215	

N, number of events.

### 3.2 Product rankings and future consideration

Participants ranked each product at several points during the study, and opinions changed substantially with increased exposure and use (Table 3).

**Table 3:** Quatro product rankings, choice and future consideration

	Ring	Insert	Film	Gel
	R%	R%	R%	R%
Ranked #1 – Most preferred^a^				
Enrolment, pre‐video (n = 200)	15	25	19	41
Enrolment, post‐video (n = 200)	**25**	25	17	33
Month 4, after crossover completed (n = 180)	**29**	26	**29**	**16**
Ranked #4 – Least preferred^a^				
Enrolment, pre‐video (n = 200)	63	9	13	15
Enrolment, post‐video (n = 200)	**53**	11	17	19
Month 4, after crossover completed (n = 180)	**42**	12	**23**	**23**
Would NOT consider using in the future^b^ (n = 180)	32	8	14	17
Choice, for use in Month 5^c^ (n = 180)	28	26	29	16
South Africa (n = 87)	28	**34**	13	**25**
Zimbabwe (n = 93)	29	18	**45**	8

R%, row percent.

^a^Estimated proportions from mixed‐effect logistic regression models adjusted for country; bold indicates significant difference from ranking pre‐video (*p *<* *0.05); ^b^could select more than one product; 56 women (31%) said they would consider using all in the future; ^c^bold indicates significant difference between countries (*p *<* *0.05).

Prior to watching the educational video at baseline, the ring was ranked least preferred (64% ranked it fourth out of the four options while only 16% ranked it as first, or most preferred). Following the video, preference for the ring increased, with 26% ranking it first and 53% as fourth (*p *=* *0.01). After using all four products during the crossover period, nearly equal proportions of women ranked the ring (29%), film (29%) or insert (26%) as their most preferred product; 16% ranked the gel most preferred. The gel followed an opposite pattern to the ring: a substantial proportion (41%) ranked the gel as #1 most preferred prior to the video, whereas the smallest proportion ranked it #1 after use (<0.001). The monthly ring, although making a significant improvement with increased exposure and use, was least preferred by a greater proportion of women (42%) compared to any of the on‐demand products (Table 3). Product rankings differed notably by country: more Zimbabwean women ranked the film most preferred than South African women (45% vs. 13%, *p *<* *0.001). In contrast, more South African women preferred the insert (35 vs. 18%; *p *=* *0.004).

Importantly, after using each of the products during the crossover period, 81% of participants changed their mind regarding which product was their most preferred; in other words, only 19% ranked the same product as their most preferred (ranked 1) from prior to seeing the video, through Month 4.

When asked about products they would not use in the future, 32% of women said they would not use the ring. A smaller proportion reported a lack of willingness to use the gel (17%); film (14%); or insert (8%) in the future. Five participants (3%) indicated two products they would not consider in the future. Fifty‐six women (31%) indicated they would consider using all products (Table 3).

### 3.3 Choice

After the crossover period, women were asked to choose one product to use for the fifth and final month of the study. Among the 180 women who completed the crossover, 29% chose the film (95% CI: 23%, 36%), 28% chose the ring (95% CI: 22%, 35%), 26% chose the insert (95% CI: 20%, 32%), and 16% chose the gel (95% CI: 11%, 21%). Overall, 96% of women chose to use the product that they had ranked first (most preferred). As such, choice of film, insert and gel followed a similar pattern of differing significantly by country (Table 3): 45% of Zimbabwean women chose the film, versus 13% of South African women (*p *<* *0.001). In South Africa, 34% chose the insert and 25% the gel (vs. 18% and 8% respectively in Zimbabwe). There was no difference in odds of choosing the ring by country.

### 3.4 Product use

Table 4 summarizes the self‐reported use of each product overall, and during sex, for the cross‐over period and final month of the study. During the crossover period, 67% of women reported using the ring for the entire month; 84% to 87% of women reported using the insert, gel or film at least once a week. These proportions were lower during sex for all products, except the ring.

**Table 4:** Self‐reported use of products during crossover and choice periods, with and without use during sex

	South Africa				Zimbabwe				Total			
	Ring	Insert	Film	Gel	Ring	Insert	Film	Gel	Ring	Insert	Film	Gel
	%	%	%	%	%	%	%	%	%	%	%	%
Crossover period – Month 1 to 4	N = 89	N = 88	N = 89	N = 89	N = 96	N = 96	N = 97	N = 97	N = 185	N = 184	N = 186	N = 186
Used all product^a^	52	82	79	78	81	90	90	95	67	86	84	87
Used product at least once with sex^b^	71	69	64	72	96	92	95	95	84	81	80	84
Choice period – Month 5	N = 22	N = 29	N = 12	N = 22	N = 26	N = 17	N = 41	N = 7	N = 48	N = 46	N = 53	N = 29
Median number of vaginal sex acts past 30 days (IQR)	2 (1 to 4)	5 (3 to 10)	5 (4 to 8)	4 (1 to 5)	12 (10 to 20)	18 (15 to 30)	20 (15 to 25)	13 (8 to 22)	8 (2 to 13)	9 (4 to 16)	18 (7 to 24)	4 (2 to 8)
Used product most or all of the time with sex	77	48	58	55	96	53	37	57	88	50	42	55
Used product some of the time with sex	5	48	42	27	4	47	56	43	4	48	53	31
No sex in past 30 days	18	3	0	18	0	0	7	0	8	2	6	14

IQR, interquartile range.

^a^Used four times or ring for 28 days/entire visit period; ^b^participants were not required to use with sex during crossover period.

Self‐reported product use during the final month of the study varied importantly by product type. For the 48 women using the ring, 88% reported using the ring “most or all of the time” they had sex. By contrast, self‐reported use during sex for the on‐demand products was inconsistent, with proportions ranging from 42% to 55% reporting use “most or all of the time” during sex.

### 3.5 Predictors of choice

Logistic regression models (one for each product) were used to assess associations between participant demographics/ behaviour characteristics and choosing the product (vs. not) for use in Month 5 (Table 5). Each model controlled for country and crossover month of choice product use. In each of the models for the on‐demand products, country was significantly associated with choice (*p *<* *0.001). Previous experience with contraceptive implants was significantly associated with choice of the ring (AOR 2.23, 95% CI: 1.07, 5.64; *p *=* *0.03).

**Table 5:** Individual logistic regression models for each Quatro product examining associations between participant characteristics and choice of product for use in Month 5

Separate model for each of the following:	Choice product											
	Ring			Insert			Film			Gel		
	N = 180			N = 180			N = 180			N = 180		
	%	AOR	95% CI	%	AOR	95% CI	%	AOR	95% CI	%	AOR	95% CI
Country, Zimbabwe versus South Africa	57	1.07	(0.58 to 2.06)	**39**	**0.43**	**(0.21** to **0.85)**	**80**	**5.80**	**(2.71** to **12.40)**	**25**	**0.23**	**(0.09** to **0.58)**
Age group, 25 to 30 versus 18 to 24 years old	39	0.95	(0.48 to 1.86)	30	0.61	(0.29 to 1.26)	45	1.07	(0.53 to 2.16)	48	2.00	(0.85 to 4.72)
Married or living with partner	53	0.56	(0.15 to 2.12)	43	1.31	(0.37 to 4.67)	81	2.34	(0.59 to 9.30)	28	0.55	(0.11 to 2.60)
Parous	88	1.30	(0.44 to 3.85)	85	1.31	(0.47 to 3.69)	96	1.65	(0.33 to 8.33)	69	0.48	(0.17 to 1.37)
Completed secondary school	73	1.33	(0.64 to 2.76)	70	0.99	(0.47 to 2.09)	62	0.87	(0.42 to 1.80)	69	0.77	(0.31 to 1.91)
Earns income	41	1.57	(0.75 to 3.28)	23	0.60	(0.26 to 1.37)	42	0.72	(0.34 to 1.53)	31	1.74	(0.64 to 4.74)
Never/rarely attends religious service	12	0.62	(0.21 to 1.80)	21	1.08	(0.42 to 2.79)	6	0.77	(0.19 to 3.16)	31	1.71	(0.62 to 4.72)
Has place for privacy in home	96	1.78	(0.37 to 8.62)	91	0.69	(0.19 to 2.56)	94	0.85	(0.19 to 3.71)	93	0.96	(0.19 to 4.96)
Food worry past four weeks	31	1.09	(0.52 to 2.32)	30	0.66	(0.30 to 1.45)	25	1.33	(0.56 to 3.12)	41	1.11	(0.46 to 2.70)
Worried about HIV	49	0.79	(0.41 to 1.52)	57	1.55	(0.77 to 3.11)	47	0.64	(0.31 to 1.29)	59	1.50	(0.64 to 3.49)
Any intravaginal practices past three months	84	0.75	(0.30 to 1.91)	85	0.99	(0.37 to 2.63)	91	1.20	(0.39 to 3.71)	86	1.28	(0.39 to 4.26)
Contraceptive methods ever used												
Male condom	80	1.55	(0.65 to 3.70)	79	0.75	(0.30 to 1.89)	58	0.51	(0.23 to 1.12)	97	6.35	(0.79 to 51.34)
Implants	**45**	**2.24**	**(1.08** to **4.66)**	21	0.61	(0.27 to 1.40)	36	0.69	(0.32 to 1.47)	24	0.98	(0.36 to 2.70)
Injectable	51	1.08	(0.54 to 2.18)	62	1.50	(0.72 to 3.12)	38	0.87	(0.41 to 1.81)	52	0.59	(0.24 to 1.43)
Pills	61	0.93	(0.41 to 2.12)	53	1.16	(0.51 to 2.67)	75	0.94	(0.37 to 2.42)	45	0.95	(0.36 to 2.50)
Knew about product at enrolment	55	2.30	(0.59 to 8.98)	23	1.12	(0.37 to 3.35)	49	0.63	(0.26 to 1.53)	21	0.38	(0.10 to 1.39)
Told partner about product during crossover	75	1.11	(0.48 to 2.56)	64	1.06	(0.48 to 2.33)	72	0.44	(0.16 to 1.23)	62	1.78	(0.69 to 4.58)
Sex during crossover month, >4 times	65	1.51	(0.54 to 4.28)	50	1.55	(0.56 to 4.29)	77	1.23	(0.43 to 3.52)	31	0.48	(0.16 to 1.45)

All models controlled for country and crossover month of use. Bold text indicates significance at *p *<* *0.05. AOR, adjusted odds ratio; CI, confidence interval.

## 4 Discussion

In this study, four vaginal placebo dosage forms for HIV prevention were investigated among young Zimbabwean and South African potential end‐users. The results provide three important insights into the preferences and use experiences of these women. First, preferences varied within and across countries – there was no clear favourite – indicating the need for a range of options for end‐users. Second, opinions shifted after participants learned more about a product, and after they tried it a few times, demonstrating key steps in the adoption of a novel product. Finally, the relationship between acceptability of, and adherence to, a product is not straightforward: while the ring was not necessarily the most favoured overall and consistency of use during the crossover period was lower than other methods, it was the most consistently used product among women who chose it as their preferred dosage form.

Just under half of the women in Zimbabwe chose the vaginal film and ranked it their most favoured product, whereas in South Africa the film was the least chosen form, and the vaginal insert was the most favoured, by just over one‐third of women. In both settings, the ring was the second most favoured and chosen product, but also the most likely to be ranked last (least preferred). When controlling for other demographic and behavioural factors, study country was highly predictive of choice for the on‐demand products. The reasons for the country differences in preference are unclear and may be related to the demographic differences in the populations, cultural perceptions or practices, prevalence of a similar delivery methodology in a community, or other factors. Differences in product choice across geographical settings have been identified in other studies. For example, in a companion study by our research group (“TRIO”), the majority of women in both Kenya and South Africa preferred injections overall, but the proportion of women in Kenya preferring oral tablets was significantly higher than in South Africa 17, 18. The findings of both studies highlight the importance of conducting multisite research to assess the generalizability of findings across settings. Furthermore, the fact that there were country differences, and that the distribution of products chosen and ranked number one was fairly evenly distributed support the findings of other research studies 17, 18, 19, 20 and the call for continued research and development of a range of options for women, a priority that has been threatened by funding cuts, fears of expensive registration trials, and false assumptions about what methods women will use 21, 22. The contraceptive field can offer important parallels: as the method mix for modern contraception has increased in Sub‐Saharan Africa, so has the overall proportion of users 23, 24.

In this study, product ranking for the ring shifted in a positive direction following information received through a short animated video, and then further increased through actual use. Although this study was not designed to measure a specific behavioural framework, the observed trajectory is reflective of a stages of change model 25, 26, which describes behavioural transitions from knowledge to action; and models based on the theory of diffusion of innovations 27 which similarly describe the process through which new innovations, such as these novel vaginal products, are accepted at a societal level. Research with participants from clinical trials of the dapivirine ring has highlighted that this novel technology required several months of use to build comfort and to dispel fears and concerns 10. Peer and research staff support were critical for overcoming this early adoption period. Similar results were reported among adolescent users of the contraceptive NuvaRing in the United States 28. Other studies in Africa have likewise identified that familiarity with a delivery method is correlated to preference 19. Product developers and researchers should therefore be mindful that introduction of a novel method may face initial resistance, but these opinions may be amenable to change through provision of information, support and practice in use of the method through introductory use periods. In models examining predictors of choosing products, previous experience with the implant was associated with choosing the ring. Although speculative, it is possible that the women who were more comfortable with the relatively new technological approach of inserting a contraceptive implant were similarly more comfortable with the least known formulation of a monthly inserted ring – in other words, these women were perhaps “early adopters” of novel technologies and/or product delivery approaches.

The ring was chosen by approximately 28% of participants in both countries and self‐reported adherence was substantially higher with the ring than the on‐demand products. That said, it was also the least preferred by the highest proportion (42%), suggesting a polarization of opinions around the ring. The insert and the film were the most preferred products in South African and Zimbabwe respectively, but overall only about half of those who chose these on‐demand products reportedly used them “most or all of the time” during sex. Thus, there was not a straightforward, linear relationship between preference and adherence, and the concept that preference and adherence are not necessarily linked has been discussed and reported by others 29. Nevertheless, the finding that women in Zimbabwe significantly favoured the film, and that women in South Africa significantly favoured an insert, yet in both cases, did not use them consistently during sex, warrants further investigation into how much product‐specific and contextual factors (combined with the placebo indication) contributed to their inconsistent use. Here the data suggest that although women did not prefer the ring, they used the ring more consistently during exposure to sex, presumably because it was inserted once monthly instead of needing to be inserted with every sex act. From a public health perspective, a positive attribute of the ring is that adherence is passive (does not require any action once in place) rather than active insertion as the on‐demand products require. In the HPTN 067 ADAPT study, both study arms (daily vs. intermittent oral PrEP) required active adherence but those randomized to use PrEP “on‐demand” were less adherent and had higher HIV acquisition 30. Further research with active products of differing dosage and durations, for example, the planned MTN 034 REACH study of oral PrEP and the dapivirine ring, will provide greater insight into how evidence of different delivery approaches can shape recommendations for prevention method use at an individual and population‐level.

The four products tested in this study were novel product delivery forms. In contrast to TRIO that reported a strong preference for injections over tablets and the ring, here women had a variety of preferences, demonstrating the importance of providing a range of options to women. Although one‐third of women expressed a disinterest in ever using the ring again, another approximately one‐third expressed a willingness to consider all of these product forms in the future; again endorsing the need for development of multiple dosage delivery forms.

There are some limitations to note in this analysis. Product preference and self‐reported use data may have been subject to social desirability bias. We attempted to mitigate this bias through enlistment of participants as co‐designers from the onset of the study, and reemphasizing their key role in giving candid feedback, even if it was critical of the products. Frequent report of product dislikes and nonuse during Month 5 suggest this strategy was at least partially achieved. We used placebo delivery forms in this study to focus on the delivery form in isolation from any drug‐related (side) effects. Results may have been different with the use of active products.

## 5 Conclusions

In conclusion, the Quatro data showed that young women's preferences for four vaginally‐inserted methods varied by product – there was no dominant favourite; they varied before and after use, and they varied by country. The results highlight the importance of conducting research with end‐users during the early stage of product development, ideally in a participatory manner that enables constructive feedback. As in other fields of prevention, it is unlikely that there will be a “silver bullet” for HIV. These study results reinforce, and provide evidence for the importance of providing women with a choice of options for HIV prevention.

## Competing interests

All authors confirm that they have no conflicts of interest to declare.

## Authors’ contributions

All authors contributed to the interpretation of the data and reviewed and edited the manuscript. ETM and EB led the writing and development of the manuscript. PM, NMM, ZMC, MB, NM, MJ and JS were involved with site implementation of the study protocol. RW, SJ, PM, NM and MJ oversaw study implementation procedures, data collection and study management. ETM, MB, JS1, AVS, JS2, ZMC and GFD designed the study and provided scientific oversight to the design of the study protocol, data collection instruments and procedures. EB led the statistical analyses.
